# Structures of Mec1/ATR kinase endogenously stimulated by different genotoxins

**DOI:** 10.1038/s41421-022-00461-8

**Published:** 2022-09-29

**Authors:** Qingjun Zhang, Po Wang, Tengwei Wu, Yueyue Zhang, Zexuan Zheng, Shangzhi Zhou, Dong Qian, Xuejuan Wang, Gang Cai

**Affiliations:** 1 The First Affiliated Hospital of USTC, MOE Key Laboratory for Cellular Dynamics, Division of Life Sciences and Medicine, University of Science and Technology of China, Hefei, Anhui China; 2 Center for Excellence in Molecular Cell Science, Chinese Academy of Sciences, Hefei, Anhui China; 3 Department of Radiation Oncology, the First Affiliated Hospital of USTC, Division of Life Sciences and Medicine, University of Science and Technology of China, Hefei, Anhui China

**Keywords:** Cryoelectron microscopy, DNA damage response

Dear Editor,

The ataxia-telangiectasia mutated (ATM) and ATM-Rad3-related (ATR) are apical kinases that orchestrate the multifaceted DNA damage response to a variety of insults and regulate genomic stability^1^. Notably, in yeast and human, ATM is not essential, whereas ATR is^2^. The basal kinase activity of ATR plays critical regulatory roles during normal cell-cycle progression and aberrant activation of ATR signaling pathway can drive cells into senescence^3^. In 2017, we reported the first cryo-EM reconstruction of intact Mec1-Ddc2 (the yeast homolog of ATR-ATRIP), which elucidated how the kinase activity of Mec1 is poised for catalysis by the inhibition of the PRD domain^4^. Recently, the structure of the constitutively active mutant Mec1(F2244L)-Ddc2 complex identified the conformational changes of the active site and substantiated the PRD’s inhibition effect^5^. Still, it remains ambiguous how wide type (WT) ATR/Mec1 is activated under physiological conditions, hindering our understanding of DNA damage response processes and development of therapeutic agents targeting the kinase^6^.

Three direct Mec1 activators have been identified in yeast, which are 9-1-1 checkpoint clamp, Dpb11 (human TopBP1) and Dna2. It is impossible to recapitulate the in vivo activation process using any specific activator alone. Therefore, we prefer to induce endogenous DNA damages to activate Mec1 in vivo for structural analysis. Hydroxyurea (HU) and methylmethane sulfonate (MMS) are utilized to induce replication- and genotoxic-stress on the cultured yeast cells. HU inhibits dNTP production and slows replication forks globally, while MMS mainly methylates purines and creates polymerase-stalling lesions^7^. We endogenously purified the in vivo activated Mec1-Ddc2 (Supplementary Fig. S1a) and ran the in vitro kinase assay (Supplementary Figs. S1c and S2a). Both the HU and MMS activated ATR/Mec1 increasing kinase activity by 10-fold (Fig. 1b), which could be only marginally stimulated by the Dpb11 activator (Supplementary Fig. S2b), in agreement with previous studies with *mec1-F2244L* mutant^5^.

**Fig. 1:**
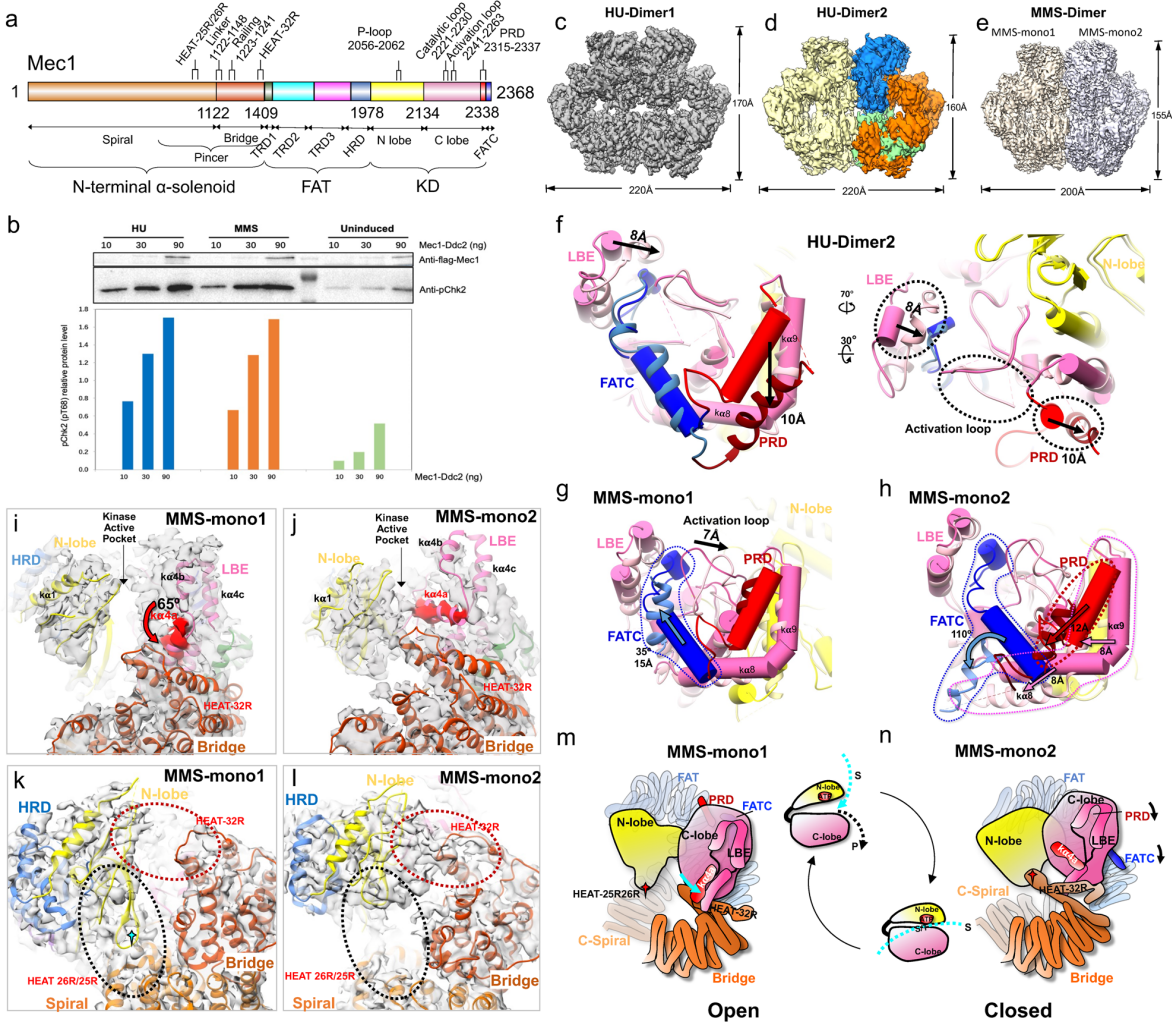
Structures of the yeast Mec1-Ddc2 complex, a homolog of human ATR-ATRIP, endogenously activated by HU and MMS. **a** Schematic representation highlighting the functional domains of Mec1/ATR kinase. Three units of Mec1 are labeled: The N-terminal α-solenoid (Spiral and Bridge), the FAT (TRD1, 2, 3 and HRD); and the KD (N-lobe, C-lobe and its PRD and FATC). The same color scheme is used in all structure figures. **b** In vitro kinase assay of the purified Mec1-Ddc2 complex. Bar representations of the relative kinase activities. **c** Front view of the density map of the HU-Dimer1. **d** Front view of the density map of the HU-Dimer2. One monomer is color-coded by domain assignment: FAT-KD-PRD-FATC in blue, N-terminal α-solenoid in orange, and Ddc2 in light green. The other monomer is shown as a solid yellow surface. **e** Front view of the density map of the MMS-Dimer, with the two asymmetric monomers differently colored. **f** Comparison of the active site structure of the HU-Dimer2 (shown in ribbons) and that of the Mec1 with basal activity state (shown as transparent pipes). **g**, **h** Comparison of the active site structure of the active site of MMS-mono1 and MMS-mono2 (shown in ribbons) with that of the Mec1 harboring basal activity state (shown as transparent pipes). **i**-**l** Close-up views of the model-map fitting of the intramolecular interfaces of the MMS activated Mec1. **i, j** highlights the conformational variation of the LBE with the motion range labeled. **k**, **l** highlights the N-lobe alternatively docks with C-terminal of the Spiral (denoted by a black dashed oval) or with the Bridge domain (denoted by a red dashed oval). **m**, **n** A structure-based model illustrating the N-lobe of activated Mec1 kinase alternately packs against the C-terminal of the Spiral or the Bridge, leading to the active site in wide open or tight closed conformations. The LBE Kα4a and Bridge HEAT-32R could function as two gates blocking kinase active centers.

Firstly, we determined the cryo-EM structure of Mec1-Ddc2 activated by HU (Supplementary Fig. S3). About 40% of particles have been classified into the published conformational state harboring basal activity^4^. Although the global resolution is only 4.0 angstroms (HU-Dimer1, Supplementary Table S1), some regions, such as HRD region, actually show better side chain densities (Fig. 1a, c; Supplementary Fig. S3i). The HRD packs with both N and C lobes adjacent to the kinase catalytic cleft and the improved HRD density may indicate the stabilizing effect of HRD on kinase active site. About 30% particles are classified into another conformation with a global resolution of 4.5 angstroms (Fig. 1d; Supplementary Table S1). The overall height has been reduced from 170 Å (HU-Dimer1) to 160 Å (HU-Dimer2) (Supplementary Fig. S4). In the HU-Dimer2 state, the N-terminal α-solenoid and FAT domains are rather stable (Supplementary Fig. S5). The conformational changes of HU-Dimer2 are condensed in kinase domain, mainly reflected in PRD, FATC and LBE. PRD moves about 10 angstroms outward and downward (away from the activation loop), LBE moves about 8 angstroms inward, and the activation loop has room to move outward (Fig. 1f; Supplementary Fig. S5). The subtle conformational changes of TRD3 make space and enables the motion of PRD (Supplementary Fig. S5). Globally, the conformational changes of Mec1-Ddc2 activated by HU treatment, which are confined in the FATKIN (FAT-Kinase domains) region, are not significant. This is reminiscent of Mec1(F2244L)-Ddc2 mutant^5^. The Mec1 activation induced by HU treatment involves the same mechanistic step as the constitutive active Mec1(F2244L) mutant, with relocation of PRD, FATC, and LBE is coupled to subtle conformational changes in FAT and N-lobe.

We next determined the structure of Mec1-Ddc2 complex endogenously activated by MMS (Fig. 1e; Supplementary Figs. S1b, S6 and Table S1). Besides DNA bases, the alkylative stress caused by MMS also include RNA and protein adduction. The mechanism through which MMS induces DNA damages are complicated and have not been fully understood. We speculate that HU and MMS treatment may lead to different conformational changes in Mec1. Only one state could be stably classified with a resolution of 4.0 Å. The assembly adopts an asymmetric conformation despite having two identical Mec1-Ddc2 heterodimers. The whole molecule is significantly compressed, with the length and height have been reduced to 200 Å and 155 Å, respectively (Fig. 1e; Supplementary Fig. S7). We refer two monomers as ‘MMS-mono1’ and ‘MMS-mono2’, which demonstrate similar conformational changes at the domain level. After the Pincer and C-lobe are superimposed with each other, the FAT and kinase N-lobe move down by ~10 Å, whereas the Spiral of α-solenoid moves upwards by ~11 Å relative to their positions in the structure with the basal activity (Supplementary Fig. S7d). The overall structures of the two monomers are also compared. The structures of Ddc2 and Mec1 Spiral domain are well matched, but the conformations of Bridge and FATKIN are significantly different, especially the significant displacement of LBE (Supplementary Fig. S7e, f). All PIKKs also contain a LBE insertion in the kinase C-lobe and the mTOR LBE interacts with mLST8 regulator and restricts substrate entry into the active site^8^. The conformation of MMS-mono1’s substrate binding groove is largely intact. FATC rotates inward by 35 degrees and shifts upward by 15 angstroms. The activation loop translates about 7 angstroms towards the PRD (Fig. 1g; Supplementary Fig. S8a, c). In contrast, there are synergistic conformational changes of FATC, PRD, Kα9 and Kα8 in MMS-mono2. FATC rotates 110 degrees downward, coupled with the displacement of PRD Kα10 along with Kα9 and Kα8 downward to the left in the range of 8~12 Å, which more significantly exposes the activation loop to facilitate substrate binding and catalysis (Fig. 1h; Supplementary Figs. S8b, d and S9).

The most significant conformational divergence between the two monomers is at the position of LBE Kα4a and kinase N-lobe. The position of LBE in MMS-mono2 is consistent with that of basal activity sate^4^. The LBE Kα4a is roughly parallel to HEAT-32R (residues 1334–1409) (Fig. 1i; Supplementary Fig. S9b). Whereas the Kα4a of LBE of MMS-mono1 deflects downward by about 65 degrees, close to being perpendicular to HEAT-32R (Fig. 1j; Supplementary Fig. S9a). In MMS-mono1, the kinase N-lobe packs against the HEAT 25 R/26 R (residues 1005–1090), in close proximity to the Ser1333 residue in human ATR (corresponding to Thr1092 in Mec1) (Fig. 1k). A point mutation of Ser1333 in ATR creates a hyperactive kinase^9^. The N-lobe docks at HEAT 25 R/26 R in MMS-mono1 or at HEAT 32 R of Bridge in MMS-mono2 (Fig. 1l). The N-lobe of activated DNA-PK is stabilized by the ABCDE cluster (aa 2609–2647), which is comparable to the Mec1/ATR Bridge in its location not only in the primary sequence but also in its structure^10^. The Bridge of MMS activated Mec1/ATR function reminiscently of the ABCDE cluster of activated DNA-PK in stabilizing the kinase N-lobe. These two docking sites of the N-lobe at HEAT 25 R/26 R and HEAT 32 R of Mec1/ATR, previously named as Pincer, mediate multiple interaction with FATKIN domain and cradle the Mec1/ATR catalytic core as two pliers^4^. The pincer of mTOR (corresponding to its M-Heat) is responsible for RAPTOR binding^8^ and the pincer in SMG1 (corresponding to its Arch) directly binds SMG9^11^. Thus, the structural feature of Pincer is verified to be highly conserved, which plays a critical role in regulation of PIKK activity^4,12^.

Opening and closing of the active site is an integrated part of kinase catalytic cycle, which is achieved through the iteratively motion of N-lobe as a rigid body^13^. Realignment of the N and C lobes of the kinase domain as the key event in the activation of PIKK. The proper alignment of the N and C lobes is central to kinase activation, as residues critical for substrate binding and catalysis are distributed across the two lobes. We observed the N-lobe of Mec1/ATR kinase stably docks at the two pliers in the Pincer region from the structure of the endogenously activated Mec1-Ddc2 complex, which correspond to HEAT 25 R/26 R and HEAT 32 R of the N-terminal α-solenoid (Fig. 1m, n). The N-lobe toggles between two configurations and alternately packs against C-terminal of Spiral or the Bridge, resulting in the N-lobe immobilized as wide open or tight closed conformations relative to C-lobes, respectively. The LBE Kα4a and Bridge HEAT-32R could function as two gates plugging active center. In the open conformation state, substrates and products can freely enter and exit active centers (Fig. 1m). While in the closed state, a confined chamber between the N- and C- lobe is formed under the help of Kα4a and HEAT-32R, which is optimal for the phosphate transfer reaction (Fig. 1n).

This N-terminal helical solenoid serve as a PIKK scaffold, which is essential for binding proteins that associate with the PIKKs to regulate their activity and cellular localization^14^. Moreover, the solenoid is also directly or indirectly responsible for PIKK’s substrates recognition, recruitment and delivery into the kinase active site^4,8,11,12^. The iteratively motion of N-lobe directly contributes to opening and closing of the kinase catalytic core^13^. Besides the solenoid directly packs against the FAT and kinase C-lobe^4,12^, it has offers two docking sites for the N-lobe and stabilizes the active site in an open conformation allowing substrate binding and a closed conformation optimal for the phosphate transfer reaction (Supplementary Fig. S10). Therefore, the N-terminal helical solenoid globally and critically regulates the Mec1/ATR kinase activity.

In short, our study reveals a novel gating mechanism by which kinase N-lobe toggles between two alternative docking sites and recurrently commits the Mec1/ATR kinase for substrate recruitment, confined catalysis and product release. These activated states of Mec1/ATR may be conserved among PIKK members. Future high-resolution structural studies of highly activated PIKK will aid in understanding whether the gating mechanism is conserved to regulate kinase activity levels.

The cryo-EM maps have been deposited in the EM Databank under accession numbers: EMD-32912 (HU-Dimer2), EMD-32913(MMS-Dimer), and EMD-32931 (HU-Dimer1). The coordinates of structural models have been deposited in the Protein Data Bank under accession number: 7WZR (HU-Dimer2), 7WZW (MMS-Dimer).

## Supplementary information

Supplementary Figures and Table
