# Supplementary material for: Structures of Mec1/ATR kinase endogenously stimulated by different genotoxins
Source: Cell Discov. 2022 Sep 29;8:98. doi: 10.1038/s41421-022-00461-8 (PMC9523049; doi:10.1038/s41421-022-00461-8)
Supplement: Supplementary file 1 — Supplementary Figures and Table [file 41421_2022_461_MOESM1_ESM.pdf]

Supplementary Fig. S1

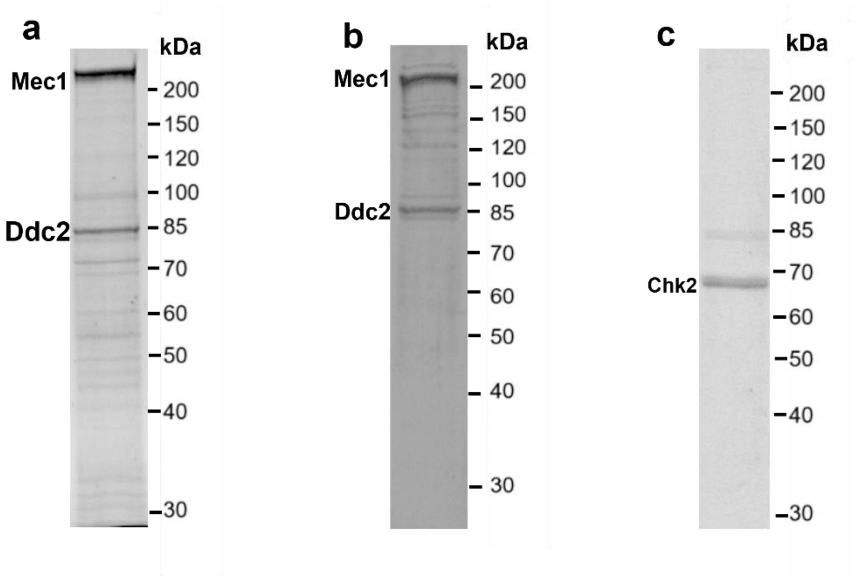

Fig. S1 Purification Mec1-Ddc2 complex activated by HU and MMS. (a). SDS-PAGE analysis of the Mec1-Ddc2 endogenously purified from *S. cerevisiae* after HU genotoxic stress. (b). SDS-PAGE analysis of the Mec1-Ddc2 endogenously purified from *S. cerevisiae* after MMS genotoxic stress. (c). SDS-PAGE analysis of the human Chk2 kinase (kinase dead mutant) recombinantly expressed from *S. cerevisiae*.

Supplementary Fig. S2

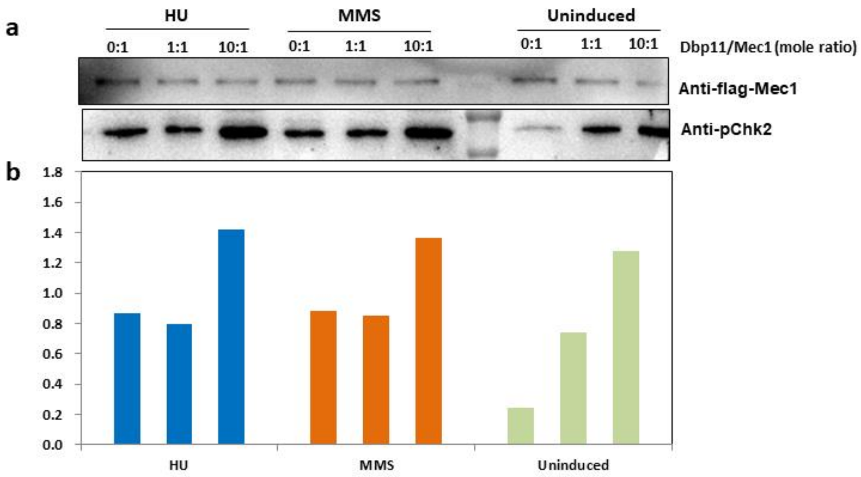

Fig. S2 *In vitro* Kinase activity assay. (a). *In vitro* kinase assay using different amount of the Mec1 activator - Dpb11 to activate the endogenously purified Mec1-Ddc2 complex. The purified kinase-dead human Chk2 (500ng) serves as substrate. The reaction was subjected to SDS–PAGE and visualized by immunoblotting with antibody specifically targeting phospho-Thr-68 of Chk2. (b). Bar representations of the relative kinase activities quantitated according to the immunoblotting results.

Supplementary Fig. S3

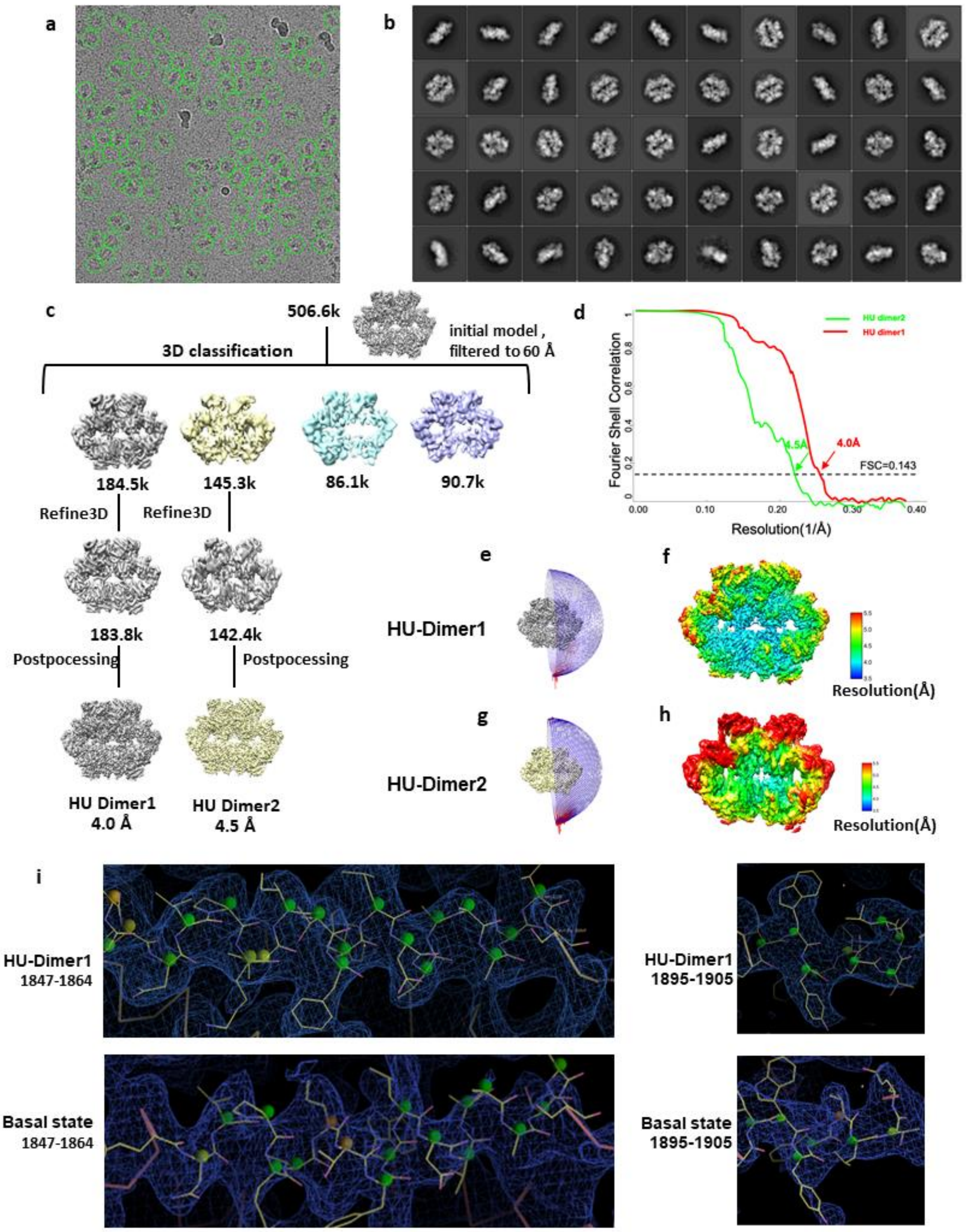

Fig. S3 Cryo-EM reconstruction of the Mec1-Ddc2 complex endogenously activated by HU. (a). A typical micrograph of Mec1-Ddc2 preserved in vitrified ice. (b). Typical 2D class averages obtained after reference-free alignment and classification of images of cryo-EM particles. (c). Cryo-EM reconstruction pipeline. The schematic diagram of the procedures is shown. The 4.0 Å map (left, HU-Dimer1, ~40% particles) closely resembles our published Mec1-Ddc2 structure harboring basal activity. However, the 4.5 Å map (right, HU-Dimer2, ~30% particles) contains substantial conformational changes in the active site. (d) FSC curves for the cryo-EM density maps according to the gold-standard criterion. (e). Angular distribution for the reconstruction of the HU-Dimer1**.** Each cylinder represents one view and the height of the cylinder is proportional to the number of particles for that view. (f). 3D density map of the HU-Dimer1 colored according to local resolution. (g). Angular distribution for the reconstruction of the HU-Dimer2**.** (h). 3D density map of the HU-Dimer2 colored according to local resolution. (i). HRD side chain densities are better defined in the EM map of HU-Dimer1 than that of the state harboring basal activity.

Supplementary Fig. S4

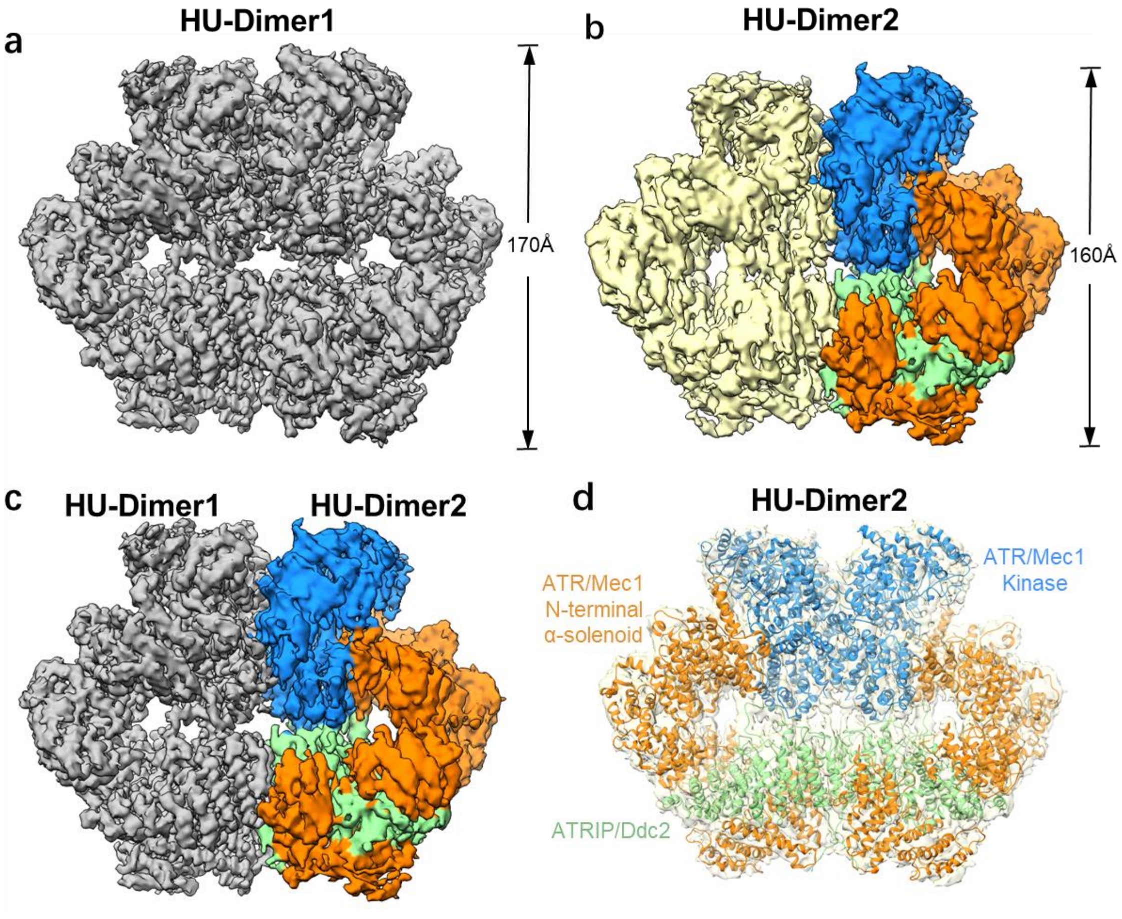

Fig. S4 Architecture of Mec1-Ddc2 induced by HU. (a) Front views of the density map of Mec1-Ddc2 in HU-Dimer1 state. (b) Front view of the Mec1-Ddc2 in HU-Dimer2 state. One of the monomers is colored as FAT-KD-PRD-FATC in blue, N-terminal a-solenoid in orange, and Ddc2 in light green. (c) Side by side comparison between two monomers respectively from HU-Dimer1 and HU-Dimer2. (d) The ribbon diagram model of the Mec1-Ddc2 in HU-Dimer2 state.

Supplementary Fig. S5

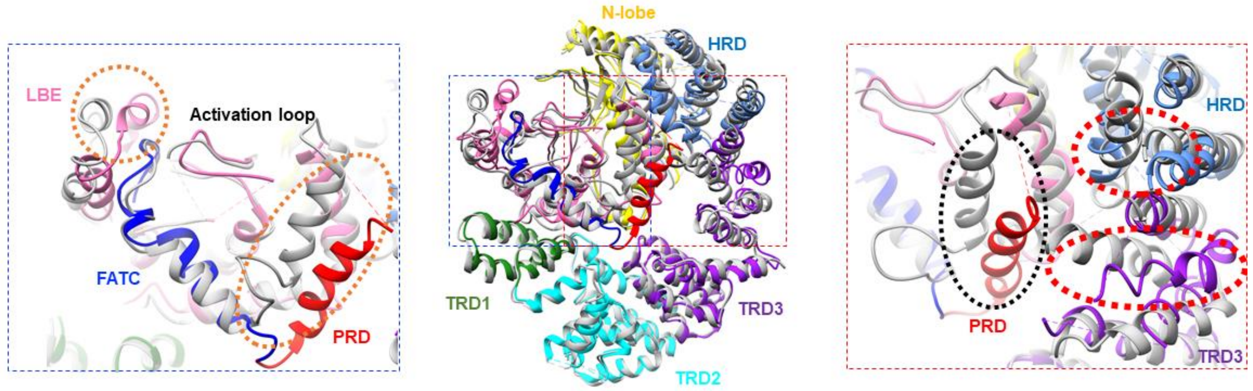

Fig. S5 Three close-up views of the model-map fitting in FATKIN domain of Mec1 activated by HU. Superimposition of the FATKIN of Mec1-Ddc2 of HU-Dimer2 (colored by domain as Fig 1a) and that of basal activity state (shown in gray).

Supplementary Fig. S6

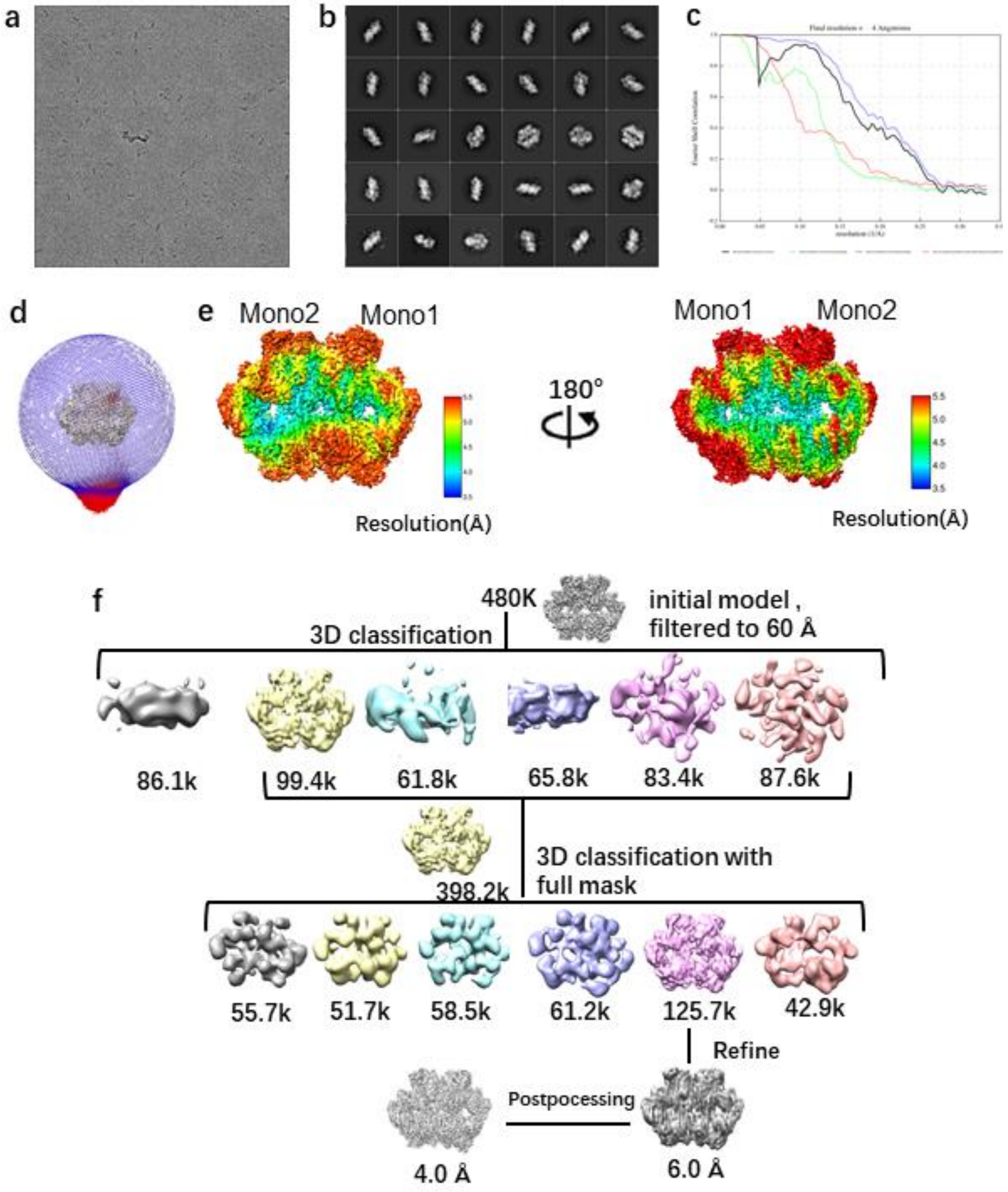

Fig. S6 Cryo-EM reconstruction of the Mec1-Ddc2 complex endogenously activated by MMS. (a). A typical micrograph of Mec1-Ddc2 preserved in vitrified ice. (b). Typical 2D class averages obtained after reference-free alignment and classification. (c). FSC curve for the cryo-EM density map according to the

gold-standard criterion. (d) Angular distribution for the reconstruction of the Mec1-Ddc2 activated by MMS**.** Each cylinder represents one view and the height of the cylinder is proportional to the number of particles for that view. FSC curve for the cryo-EM density map according to the gold-standard criterion. (e). Two views of the 3D density map colored based on local resolution, which demonstrate the differences of the two monomers in the domain mobility. (f). Cryo-EM reconstruction pipeline. The schematic diagram of the procedures is shown. Details are provided in the Method section.

Supplementary Fig. S7

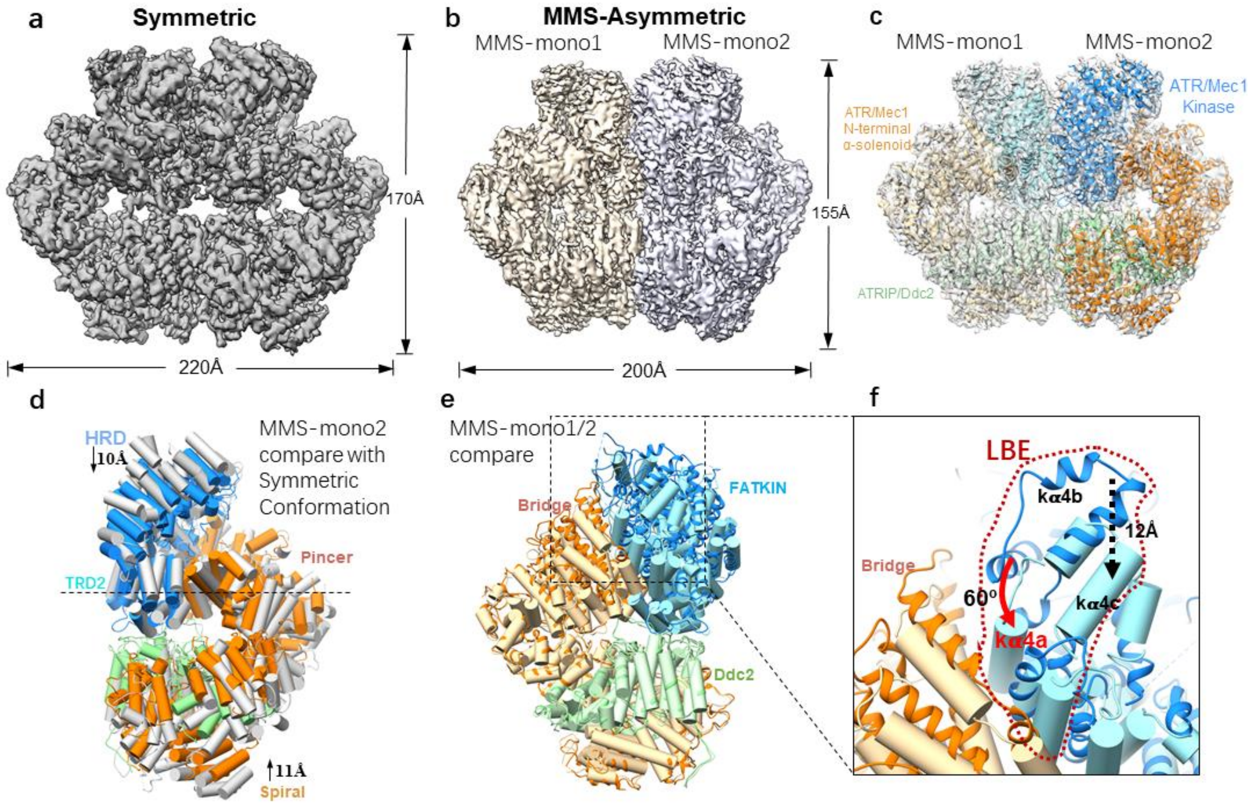

Fig. S7 Architecture of Mec1-Ddc2 activated by MMS. (a) Front views of the EM map of the symmetric homodimer of Mec1-Ddc2 in basal activity state (EMD-6708). (b) The EM map of asymmetric Mec1-Ddc2 homodimer induced by MMS with the two monomers are differently colored. (c) Front views of the Mec1-Ddc2 homodimer induced by MMS. The cryo-EM density is shown as a translucent surface and fitted with the ribbon diagram model. MMS-mono2 is color-coded by domain assignment: FAT-KD-PRD-FATC in blue, N-terminal a-solenoid in orange, and Ddc2 in light green. The MMS-mono1 is in less saturated color than the MMS-mono2. (d) Superimposition of the Mec1-Ddc2 monomer structures of the MMS-mono2 and that of basal activity state (shown in gray). (e) Overlay of the monomer structures of MMS-mono2 (shown as pipes) and MMS-mono1(shown in ribbons). (f) The LBE harbors the most substantial conformational divergences between the two monomers of Mec1-Ddc2 activated by MMS.

Supplementary Fig. S8

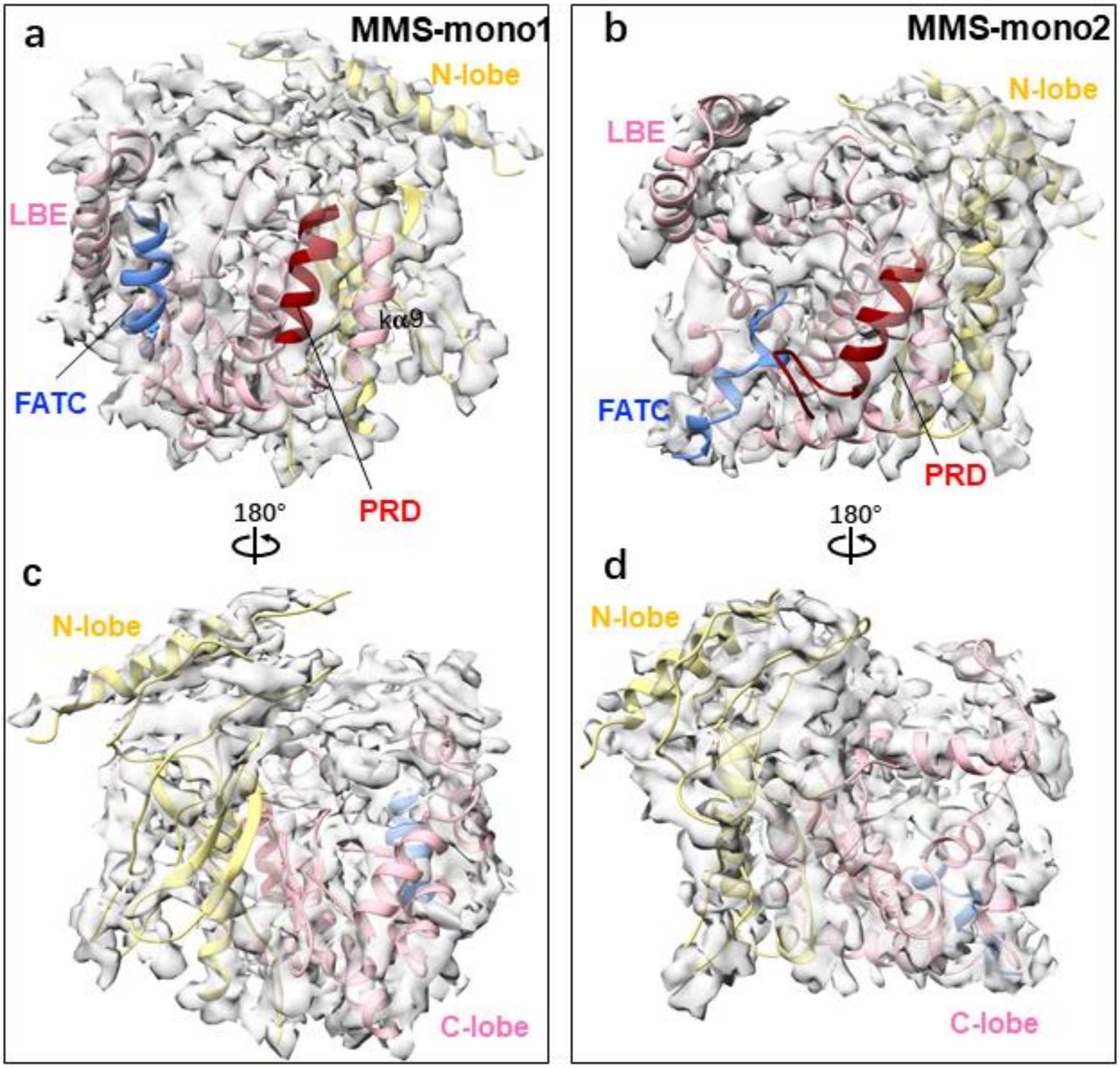

Fig. S8 The close-up views of the model-map fitting of the substrate binding groove. (a and c). Close-up views of the active site of MMS-mono1. (b and d). Close-up views of the active site of MMS-mono2.

Supplementary Fig. S9

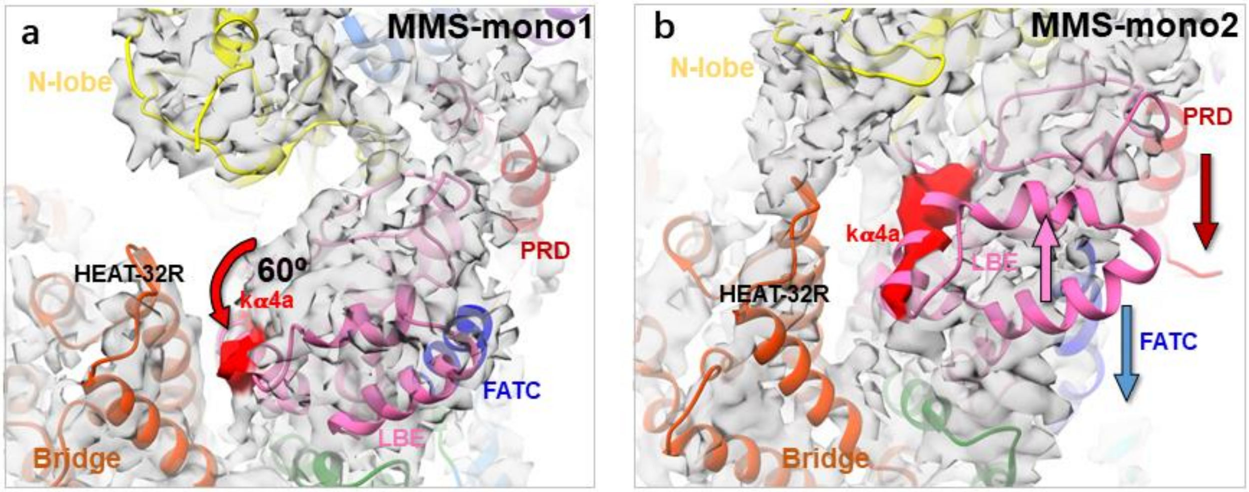

Fig. S9 The close-up views of the model-map fitting of the intramolecular interfaces between Bridge and FATKIN domains. (a). Close-up views of MMS-mono1. (b). Close-up views of the MMS-mono2 (Related to Fig. 2b). The mobility of the LBE, FATC and PRD are highlighted.

Supplementary Fig. S10

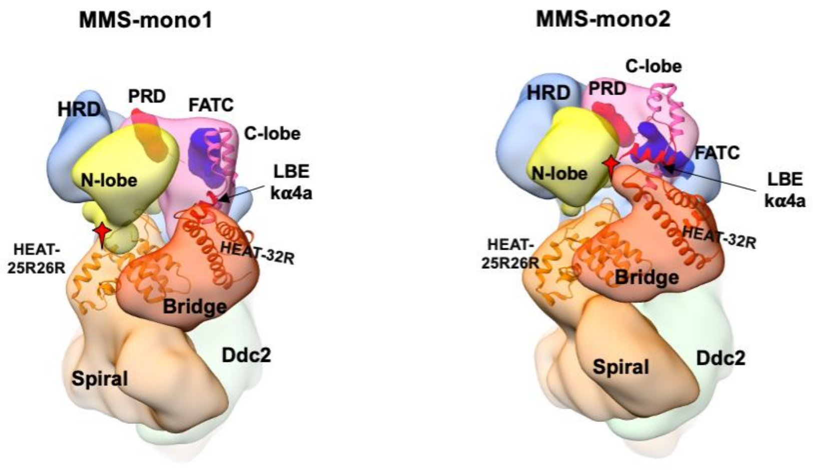

Fig. S10 Rendering of the MMS-mono1 (left) and MMS-mono2 (right) of the Mec1-Ddc2 structures (colored as in Fig. 1i-l). The structural model illustrating the N-lobe of activated Mec1 kinase alternately packs against the C-terminal of the Spiral or the Bridge, leading to the active site in wide open or tight closed conformations. The LBE Kα4a and Bridge HEAT-32R could function as two gates blocking kinase active centers. The two alternative N-lobe docking sites are highlighted with red stars.

Supplementary Table S1. Statistics of 3D reconstruction and model refinement

	MMS-Mec1-Ddc2	HU-Mec1-Ddc2 (asymmetric)	HU-Mec1-Ddc2 (symmetric)
**Data collection**			
EM equipment	Titan Krios	Titan Krios	Titan Krios
Voltage(kV)	300	300	300
Detector	Gatan K2	Gatan K2	Gatan K2
Pixel size (Å)	1.5	1.35	1.35
Electron dose (e/Å)	56	59	59
Defocus range (μm)	-2.5--3.5	-2.8--3.5	-2.8--3.5
**Reconstruction**			
Software	RELION 3.1 (beta)	RELION 3.1 (beta)	RELION 3.1 (beta)
Number of total particles	480k	506.6k	506.6k
Number of used Particles	120k	183.8k	142.4k
Symmetry	C1	C1	C2
Final Resolution (Å)	4.0	4.5	4.0
Map sharpening B-factor (Å2)	-145.86	-205.81	-192.87
**Model building**			
Software	Chimera coot	Chimera coot	
**Refinement**			
Software	phenix	phenix	
**Validation**			
Bonds length (Å)	0.009	0.009	
Bonds Angles (°)	1.278	1.213	
**Ramachandran plot statistics (%)**			
Preferred	80.26	76.29	
Allowed	18.58	22.63	
Qutlier	1.06	1.08	

Materials and Methods

Purification of Yeast Mec1-Ddc2

S. cerevisiae stain (*MATa Tel1-FLAG his3△1 leu2△0 met15 △0 ura3△0)* [1] was grown in 20L fermenter to the stationary phase. 0.05% MMS was added and 0.9kg cells were collected 90min later. Cells were washed and re-suspended in extraction buffer (50 mM HEPES [pH 7.6], 300 mM KOAc, 0.5 mM EDTA, 5 mM β-ME, 10% (v/v) glycerol, 0.1% (v/v) NP-40 and protease inhibitors) and a whole-cell extraction was prepared as previously described [2]. This whole-cell extract was selectively precipitated in 30%–55% ammonium sulfate and resuspended using 1 X TEZ buffer (50 mM Tris [pH 7.6], 1 mM EDTA, 10 uM $ZnCl_2$, 10% (v/v) glycerol and protease inhibitors). After the suspension was clarified by centrifugation, the supernatant was incubated for 2 hours at 4 ℃ with 1 ml of a 50% slurry of FLAG resin beads (Sigma) that had been pre-equilibrated with 1 X TEZ plus 250 mM ammonium sulfate. After incubation, the beads were washed with 50 ml of 1 X TEZ plus 500 mM ammonium sulfate, followed by a second wash with 50 ml of 1 X TEZ plus 50 mM ammonium sulfate. After equilibration of the column with 1 X TEZ plus 100 mM ammonium sulfate, 10 mM 3 X FLAG peptide (Sigma) was added to the resin beads and incubated overnight at 4 ℃. The Mec1-Ddc2 fraction was then eluted with three column volumes of 1 X TEZ plus 100 mM ammonium sulfate and the resulting aliquot was snap-frozen in liquid nitrogen and temporarily stored at -80 ℃. For the next purification step, the FLAG elute fractions were thawed in ice and applied onto a Mono S column (GE Healthcare) in S100 buffer (1 X TEZ plus 100 mM ammonium sulfate, 0.02% NP-40, 10 mM β-ME, 4% (v/v) glycerol) and was resolved over a 100-1000 mM ammonium sulfate gradient. The Mec1-Ddc2 elution was flash-frozen in liquid nitrogen, analyzed by SDS-PAGE and EM examination.

Kinase activity assay

To compare the kinase activities of Mec1-Ddc2 preparations induced by different genotoxic agents and eliminate contributions by substrate kinase itself, we performed an in vitro kinase assay using the purified human Chk2 (kinase dead mutant, referred to as Chk2 below) as a representative substrate. The ORF of kinase-dead Chk2 (K249R) [3] was cloned into a p416 vector and overexpressed in the protease-deficient yeast strain BJ2168 (*MATa leu2 trp1 ura3-52 prb1-1122 pep4-3 prc1-407 gal2)*. The Purification of Chk2 was the same as previously described. The ORF of Dpb11 was cloned into a pbl512 vector and overexpressed in the protease-deficient yeast strain BJ2168 (*MATa leu2 trp1 ura3-52 prb1-1122 pep4-3 prc1-407 gal2)*. The purification of Dpb11 was the same as previously described.

Kinase assays were performed in a 20 ul volume of 50mM HEPES [pH7.6], 100mM ammonium sulfate, 10% (v/v) glycerol,10μM $ZnCl_2$, 5 mM β mercaptoethanol, 4mM magnesium acetate (final concentration, including contributions made by protein storage buffers), 1mM ATP. 500ng Chk2 was incubated with Mec1-Ddc2 in 20 μl reaction buffer at 30 ℃ for 90 min. Reactions were terminated by the addition of SDS-PAGE sample loading buffer and analyzed by western blotting. Western blotting results were quantified by ImageJ program.

Sample vitrification and Cryo-EM data collection

Negative staining was used to evaluate the protein quality. In brief, 3μl of purified Mec1 was applied onto copper grids supported by a thin layer of glow-discharged carbon film (Zhongjingkeyi Technology). After adsorption for 30s, uranyl formate 0.75 % (w/w) was used for negative staining at room temperature. The negative stained grid was examined using Tecnai F12 electron microscope (FEI).

Samples were diluted to final concentration 20-50 μg/ml (20mM HEPES [pH8.0], 40mM KOAc, 5mM $MgCl_2$, 0.1% trehalose, 2mM DTT, 0.01% NP-40) and 3μl of aliquots were applied to freshly glow discharged lacey grids coated with a second layer of thin carbon film. The grids were blotted for 3 to 4s at 4 ℃ in 100% humility, then plunged into liquid ethane using an EMGP2 (Leica company). Frozen grids were stored in liquid nitrogen.

Mec1-Ddc2-MMS Cryo-EM data were collected at 300 KeV acceleration voltage on Titan Krios microscope, images were recorded on a K2 direct electron detector at a nominal magnification of 18000 with a defocus range of 2.3 μm to 3.5 μm, resulting in a calibrated sampling of 1.5 Å per pixel. The total accumulated dose rate was set to be 56 e^2 per $Å^2$ on the specimen and the exposure time was 12.5s. Each image was fractionated into 50 frames.

Mec1-Ddc2-HU Cryo-EM data were collected at 300 KeV acceleration voltage on Titan Krios microscope, images were recorded on a K2 direct electron detector at a nominal magnification of 18000 with a defocus range of 2.8 μm to 3.5 μm, resulting in a calibrated sampling of 1.35 Å per pixel. The total accumulated dose rate was set to be 59 e^2 per $Å^2$ on the specimen and the exposure time was 14.08s. Each image was fractionated into 32 frames.

Image processing

Frames were summed to a single micrograph for subsequent processing using motion correction procedure in Relion [4]. CTF parameters and defocus values for each micrograph were determined by using CTFFIND3 [5]. Cryolo [6] was used to pick particles. Totally, 480k Mec1-Ddc2 particles were picked after 2D classification and carefully cleaning. 3D classification and auto refinement were performed using RELION. The previous structure of Mec1-Ddc2 PDB: 5x6o was low-pass filtered to 60 Å and used as the starting model for the 3D classification. A final set of particles was subjected to 3D refinement. 3D refinement used gold-standard fourier shell correlation (FSC) calculations to avoid overfitting and reported resolution was based on the FSC 0.143 criterion. The postprocessing was carried out by "postprocess procedure" in Relion. Local resolution map was calculated using ResMap [7]. All the 3D structures were displayed by Chimera [8].

Model building and simulation

The model of Mec1-Ddc2 was generated based on the previous structure of Mec1-Ddc2 (PDBID: 6z3a) [9] and docked into the map. During this procedure we observed that some region of the model does not fit with the map very well especially kinase domain. So, the model was divided into several separate pieces. These pieces were then manually docked into the density map separately using Chimera. Real-space refinement (phenix.real_space_refine) [10] in Phenix was used for model refinement with secondary structure and stereochemical constraints applied. The refined model was then further rebuilt in Coot [11].

Supplementary references

1 Kapoor, P. *et al.* Regulation of Mec1 kinase activity by the SWI/SNF chromatin remodeling complex. *Genes & development* **29**, 591-602 (2015). https://doi.org:10.1101/gad.257626.114

2 Takagi, Y., Chadick, J. Z., Davis, J. A. & Asturias, F. J. Preponderance of free mediator in the yeast Saccharomyces cerevisiae. *The Journal of biological chemistry* **280**, 31200-31207 (2005). https://doi.org:10.1074/jbc.C500150200

3 Xiao, J. *et al.* Structural insights into the activation of ATM kinase. *Cell research* **29**, 683-685 (2019). https://doi.org:10.1038/s41422-019-0205-0

4 Scheres, S. H. RELION: implementation of a Bayesian approach to cryo-EM structure determination. *Journal of structural biology* **180**, 519-530 (2012). https://doi.org:10.1016/j.jsb.2012.09.006

5 Mindell, J. A. & Grigorieff, N. Accurate determination of local defocus and specimen tilt in electron microscopy. *Journal of structural biology* **142**, 334-347 (2003). https://doi.org:10.1016/s1047-8477(03)00069-8

6 Wagner, T. *et al.* SPHIRE-crYOLO is a fast and accurate fully automated particle picker for cryo-EM. *Commun Biol* **2**, 218 (2019). https://doi.org:10.1038/s42003-019-0437-z

7 Swint-Kruse, L. & Brown, C. S. Resmap: automated representation of macromolecular interfaces as two-dimensional networks. *Bioinformatics* **21**, 3327-3328 (2005). https://doi.org:10.1093/bioinformatics/bti511

8 Pettersen, E. F. *et al.* UCSF Chimera--a visualization system for exploratory research and analysis. *J Comput Chem* **25**, 1605-1612 (2004). https://doi.org:10.1002/jcc.20084

9 Tannous, E. A., Yates, L. A., Zhang, X. & Burgers, P. M. Mechanism of auto-inhibition and activation of Mec1(ATR) checkpoint kinase. *Nature structural & molecular biology* **28**, 50-61 (2021). https://doi.org:10.1038/s41594-020-00522-0

10 Adams, P. D. *et al.* PHENIX: a comprehensive Python-based system for macromolecular structure solution. *Acta Crystallogr D Biol Crystallogr* **66**, 213-221 (2010). https://doi.org:10.1107/S0907444909052925

11 Emsley, P., Lohkamp, B., Scott, W. G. & Cowtan, K. Features and development of Coot. *Acta Crystallogr D Biol Crystallogr* **66**, 486-501 (2010). https://doi.org:10.1107/S0907444910007493
